# Effect on genetic diversity of the absence of intraspecies preference in 2 sympatric *Reticulitermes* termite species (Isoptera: Rhinotermitidae)

**DOI:** 10.1093/jisesa/iead115

**Published:** 2023-12-07

**Authors:** Jia Wu, Ya-Nan Dong, Tong Niu, Huan Wang, Ali Hassan, Bei Du

**Affiliations:** College of Agriculture and Forestry, Longdong University, Qingyang 745000, China; School of Ecology and Environment, Northwestern Polytechnical University, Xi’an 710072, China; School of Ecology and Environment, Northwestern Polytechnical University, Xi’an 710072, China; School of Ecology and Environment, Northwestern Polytechnical University, Xi’an 710072, China; Hubei Insect Resources Utilization and Sustainable Pest Management Key Laboratory, Huazhong Agricultural University, Wuhan 430070, China; Shaanxi Institute of International Trade and Commerce, Xianyang 7120046, China

**Keywords:** termites, hybridization, assortative mating, reproductive isolation, genetic diversity

## Abstract

The recombinant genotypes that can be produced when closely related species mate improve the genetic diversity of the population. Among closely related species, the link between interspecific reproduction behaviors and genetic diversity has barely been studied. *Reticulitermes chinensis* and *R. flaviceps*, which live close to each other, were used as research subjects in our study to find out how preferring conspecifics affects reproductive behavior between species. We discovered that neither *R. chinensis* nor *R. flaviceps* displayed preference behavior for conspecifics. Males of *R. chinensis* and *R. flaviceps* chased and groomed not only intraspecific females but also interspecific females. In a brief period of time, 2 mating behaviors, intra- and interspecific mating, were also observed. There were no significant differences in the duration of each behavior (tandem, grooming, and mating) between interspecies and intraspecies partners. Moreover, genetic analysis showed both interspecific mating and intraspecific mating can produce living offspring when the 2 types of mating occur in a colony. Our findings showed that there was no obvious intraspecific preference between the 2 species of termite *Reticulitermes* when it came to tandem, grooming, and mating, which not only makes it easier for interspecific hybridization to occur but also sheds light on the genetic diversity.

## Introduction

The division of labor and caste differentiation in social insects such as termites may be prominent features that make colony members work together in search of food, defend the colony, and care for offspring. Cooperation and the division of labor ensure that everything is completed efficiently and conformably within the colony, which helps social insects gain higher competitive advantages over different animals in the territory. This may be a key reason for their ecological success (Wilson and Holldobler 2005, Khan et al. 2019). The overlapping of generations is another characteristic of social insects. The inclusive fitness of a social group favors cooperation among kin, resulting in inevitable inbreeding (Calleri et al. 2006). However, it is unclear how the genetic diversity of social insect populations is maintained prior to frequent inbreeding, particularly in an exceedingly closed colony throughout its life span without additional genetic contribution from outside once initial pair formation occurs.

As it has been learned, the presence of asexual queen succession (AQS) can avoid inbreeding and maintain genetic diversity in populations of social termites (Matsuura et al. 2009, Vargo et al. 2012, Hellemans et al. 2019). In termites, a colony is usually established by a pair of primary kings and queens (Thorne et al. 1999). The colony created by primary reproductives develops into a specific stage; the primary queen conditionally uses asexual reproduction to produce secondary queens and sexual reproduction for the production of workers and soldiers (Vargo et al. 2012, Yashiro and Matsuura 2014). Subsequently, these asexual secondary queens will mate with the primary king to avoid inbreeding between father and daughter (Matsuura et al. 2009, Kobayashi et al. 2013, Vargo 2019). Secondary queens employed by the primary queen retain the transmission rate of their genes to descendants, avoiding inbreeding and maintaining genetic diversity in the colony. However, the AQS breeding model is barely appropriate for species with the ability to undergo parthenogenesis (Nozaki et al. 2018). Only a few species of termites with parthenogenesis have been reported (Ishitani and Maekawa 2010, Peng et al. 2023). For most termites, the model (AQS) is irrelevant because the secondary reproductive progeny produced by sexual reproduction are unable to avoid inbreeding (parents–progeny), resulting in the decline of the genetic diversity of the colony.

Outbreeding was considered another mechanism for maintaining genetic diversity due to the fewer genetic relationships among colony founders. Interspecific individuals have more distant genetic relations than intraspecies individuals, so mating between interspecific individuals will produce various recombinant genotypes if hybridization can produce offspring, which contributes to the genetic diversity of the colonies (Khan et al. 2022a). Previous studies indicated that outbreeding occurs only in intraspecific individuals and is unwelcome in interspecific individuals (Khan et al. 2022b). Behavioral preferences for conspecifics might form a barrier to avoiding interaction between species, resulting in minimum chances of gene flow between species (Schumer et al. 2014, 2017). However, the preferences for conspecific partners have remarkable plasticity (Pfennig 2007, Rosenthal 2013). It is often abolished or reestablished depending on the fitness and environmental context (Seehausen et al. 1997, Fisher et al. 2006, Pfennig 2007). When preferences for conspecifics break down, it is going to facilitate hybridization between species and high genetic diversity (Rosenthal 2013).

Previous studies have reported cases of breaking down conspecific preferences in some species, such as ants (Kulmuni and Pamilo 2014), butterflies (Zhang et al. 2016), and fish (Harrison and Larson 2014). In these studies, the closely related species not only acquired the abundant genotypes produced by hybrid recombination but also maintained species independence by allowing the accumulation of other separation mechanisms (Hopkins et al. 2014). Sex-pairing pheromones of relative species are similar in termites (Aldrich and Kambhampati 2009, Hartke and Rosengaus 2011, Chouvenc et al. 2020), and neither female nor male possess externally specialized genitalia (Khan et al. 2022b). This indicated that the lack of these premating barriers may increase opportunities for heterospecific mating. In the absence of intraspecies preferences, interspecific hybridization can be a crucial mechanism for generating novel traits or increasing heterozygosity, thus enhancing or preserving genetic diversity.

The reproductive behavior of termites from swarming to mating has been described in previous studies (Wu et al. 2020, 2023). In this research study, we viewed the videos to determine the following behaviors. Tandem behavior is a part of courtship behavior for mate selection in termites: the male follows the female and antennates her abdomen as she searches for an adequate nesting site; it occurs in alates (Alates, also called an imago. They develop gradually via several nymphal instars into winged individuals.) after they fly away from their parental colony (Korb and Hartfelder 2008, Hartke and Baer 2011). A male and a female alate then establish a new colony and mate: termites copulate in the opposite position by joining their abdominal ends. During this time, male and female individuals also clean each other’s bodies, which is defined as a nuptial gift for mating (Vargo and Husseneder 2009).

In this study, the preferences and behaviors of inconspecifics in the reproductive processes from tandem to mating were measured in the 2 sympatric species: *R. flaviceps* Oshima and *R. chinensis* Snyder. Meanwhile, the genotypes of the parents and their offspring in colonies established by a couple of *R. flaviceps* Oshima and a couple of *R. chinensis* Snyder were analyzed using microsatellite loci. Our results revealed that there is no preference for same-species partners in the 2 species when intraspecific and interspecific individuals are present in the same arena, which could provide new insights into the genetic diversity and hybridization between species in the termites.

## Materials and Methods

### Termites

Closely related species, *R. flaviceps* and *R. chinensis*, are consistently recognized as the 2 distinct species on the basis of multiple criteria, including morphology, biology (Wu et al. 2020, Khan et al. 2022a), and mitochondrial genomic characteristics (Chen et al. 2014, Zhao et al. 2016), particularly in biological characteristics. In morphology characters, the color of *R. flaviceps* pronotum is yellow, but *R. chinensis* is black. The eclosion and swarming of *R. chinensis* happen in the same year and with intervals of about half a month. But for *R. flaviceps*, the eclosion happens in the first year and the swarming happens in the second year and with intervals of about 4 months. However, closely related species *R. flaviceps* and *R. chinensis* have overlapping habitats and dispersal seasons, similar sex pheromones, and reproductive behaviors. These similar ecological and biological characteristics imply that the 2 termite species have the possibility of encountering each other in the field while searching for mates and nest sites (Wu et al. 2023). In this study, 8 colonies (4 *R. chinensis* and 4 *R. flaviceps*) were collected from Shizi Hill, Wuhan City, Hubei Province, China, during the swarming season (March–April) in 2019. The parts of the nest with alates were brought to the laboratory and promoted dispersal flight using methods described in the document (Wu et al. 2020). Subsequently, female and male alates were divided via the morphological characteristics of the seventh abdominal sternite (Miyata et al. 2004, Wu et al. 2013), and the male individuals were marked using white (uni-Paint markers PX-21, Mitsubishi Pencil Company, Tokyo, Japan) on their abdomens. To avoid pre-experimental mating, the individuals of the same sex from the same species were placed in one Petri dish (Φ = 12 cm), and all the experiments commenced on the day when the colony dispersed.

### Experimental Setup

Considering the natural sympatry, the individual dealates (alates shed their wings after the nuptial flight and were called dealates as primary reproductives) from 4 *R. flaviceps* colonies and 4 *R. chinensis* colonies were used to mimic the scenarios of their encounter with each other. We observed the preference for conspecific partner processes of tandem, grooming, and mating behavior. (i) The tandem and grooming behaviors of 2 *R. flaviceps* (1 male, 1 female) and 2 *R. chinensis* (1 male, 1 female) were placed in 60-mm Petri dishes with moistened filter paper and allowed to occur unconstrained. Videos (5 min long) were taken with a high definition camera (Nikon D7000 with 60-mm lens, Tokyo, Japan) 3 times, at 10, 30, and 50 min after the establishment of the groups because the period from encountering each other to the end of tandem takes about 1 hour (Wu et al. 2020). We prepared 24 replicates in this experiment. (ii) The behavioral observations of mating of 2 *R. flaviceps* (1 male, 1 female) and 2 *R. chinensis* (1 male, 1 female) were placed into 60-mm Petri dishes with moistened filter paper. One-hour video was taken every hour and observed continuously for 12 h (a total of 6 h were recorded). We could not monitor the mating behavior of too many different colonies at the same time due to uncertainty about the exact timing of mating after the tandem. Therefore, we prepared 8 replicates for this experiment. After the filming was finished, each group (a couple of *R. flaviceps* and *R. chinensis*) was placed into a 120-ml transparent cylindrical vial (Φ = 3 cm) and conditions for feed provided moistened filter paper and pine wood at 20–26 °C in constant darkness.

### Behavioral Observation

The reproductive behaviors, including tandem, grooming, and mating, have already been described in previous studies and that were paired behaviors between the sexes, initiated by one partner and accepted by the other (Vargo and Husseneder 2009, Hartke and Baer 2011). We viewed the videos and designated males as target individuals and extracted the frequency and duration time of tandem, grooming, and mating behaviors between inter- and intraspecies in each video during 5-min observation (3 behavioral data are the same for females and males). The behavior recorded must satisfy the following conditions: (i) behavioral duration of more than 5 s and (ii) the interval time between events is more than 3 s. We used the generalized linear mixed models for our behavioral data. In the model, explanatory variable (behavioral frequency) was treated as a fixed factor and both colonies and types of behavior (interspecific or intraspecific) as random factors. All analyses were performed with SPSS v21 (IBM Corp., Armonk, NY, USA). All values were expressed as the mean ± SEM. The *P*-value of less than 0.05 was considered to be statistically significant.

### Genetic Analyses

There have been 2 situations for interspecies reproductive behavior: one is a lack of preference for conspecific partners, and the other is reproductive interference for interspecies competition without producing offspring (Liu et al. 2007). To investigate the purpose of interspecies reproductive behavior, 5 colonies (Colony ID: H_*cf*_-032806; H_*cf*_-032810; H_*cf*_-032604; H_*cf*_-032601; H_*cf*_-032705) established 2 months ago by a couple of *R. flaviceps* and a couple of *R. chinensis* were used for genetic analysis (for detail, see Table 1). DNA was extracted from each individual using a TIANamp Genomic DNA Kit (Tian Gen Biotech Co., Ltd.) according to the manufacturer’s protocol. PCR amplification of each DNA sample including microsatellite primers (see Supplementary Table S1), PCR system, and conditions have been improved based on descriptions in the document (Wu et al. 2013). There are differences in the size of the microsatellite locus in each individual, and the sexual offspring possess inherent features from their parents. So, in combination with the genotypes of dealates, genotypes of offspring are evidence to estimate their production by hybridization or conspecific mating.

**Table 1.** The number of larvae from colony establishing a couple of *R. flaviceps* and a couple of *R. chinensis* assigned to types based on 5 SSR markers

Colony ID	No. of larvae	The number of individuals assigned to type		
		Purebreed	Hybrid	Ambiguous
H_*cf*_-032806	16	5	5	6
H_*cf*_-032810	20	7	4	9
H_*cf*_-032604	14	4	3	7
H_*cf*_-032601	12	4	5	3
H_*cf*_-032705	18	12	6	0

## Results

**Fig. 1.**
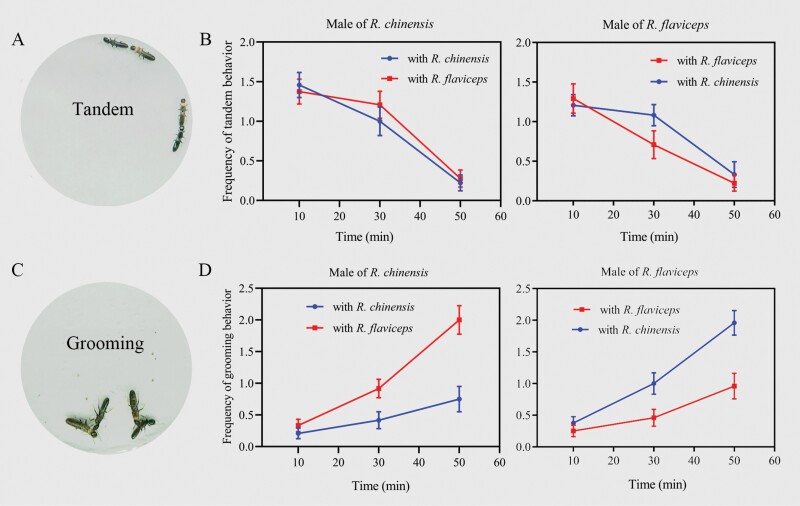
The frequencies of tandem and grooming behavior provided by male to interspecific and intraspecific female. A) Tandem behavior. B) The frequencies of tandem behavior at each observing point. C) Grooming behavior. D) The frequencies of grooming behavior at each observing point. Data are shown as means ± SE.

**Fig. 2.**
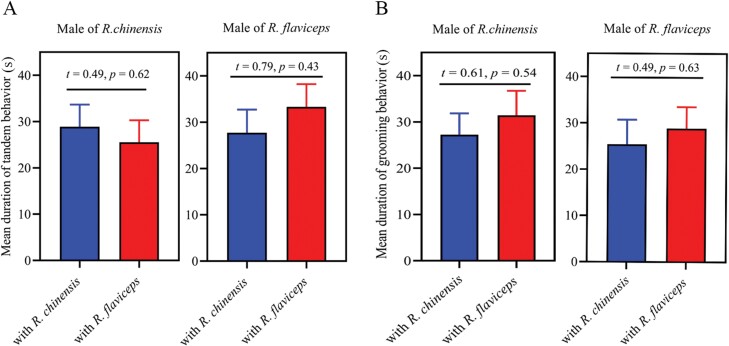
The duration of A) tandem and B) grooming in intraspecific and interspecific partners. Data are shown as means ± SE.

Males of both *R. chinensis* and *R. flaviceps* did not exhibit preference behavior for conspecific female individuals in tandem when a *R. chinensis* couple and a *R. flaviceps* couple were present in the same arena. Besides tandem with conspecifics, male of *R. chinensis* also pursued female of *R. flaviceps* vigorously (Fig. 1A). There were no significant differences in frequencies between tandem with *R. chinensis* and tandem with *R. flaviceps* during the time of observation (Fig. 1B, *F*_2,126_ = 0.21, *P* = 0.64). Male of *R. flaviceps* has similar tandem behavior to that of *R. chinensis*. The 2 tandem behavior frequencies of male *R. flaviceps*, tandem with *R. chinensis* and tandem with *R. flaviceps*, respectively, both decrease with time over, and there was a significant difference in the second observation (*t* = 1.83, *P* = 0.02), but there were no significant differences in frequencies during the total observation time (Fig. 1B; *F*_2,126_ = 0.15, *P* = 0.71). In tandem duration, there were no significant differences in 2 behaviors of male *R. chinensis*, tandem with female of *R. chinensis* and tandem female of with *R. flaviceps* (Fig. 2A; *t* = 0.49, *P* = 0.62). Similarly, 2 behaviors of male *R. flaviceps* showed no differences in duration (Fig. 2A; *t* = 0.79, *P* = 0.43). Tandem behavioral results indicated that the preferences for conspecifics were absent in *R. chinensis* and *R. flaviceps*.

Males of *R. chinensis* provided grooming not only for female of *R. chinensis* but also for female of *R. flaviceps* (Fig. 1C). Both of the grooming frequencies increased with the length of the observing time, and we surely saw a faster trend for grooming in *R. flaviceps*. Finally, the frequencies provided grooming for female of *R. flaviceps* were higher than the frequencies provided for female of *R. chinensis* (Fig. 1D; *F*_2,138_ = 17.78, *P* < 0.001). Male *R. flaviceps* groomed female *R. chinensis* more frequently than conspecific females (Fig. 1D; *F*_2,138_ = 14.57, *P* < 0.001). In grooming duration, we found no significant effect on species of female grooming provided by *R. chinensis* (Fig. 2B; *t* = 0.61, *P *= 0.54) and by the male of *R. flaviceps* (Fig. 2B; *t* = 0.49, *P* = 0.63).

**Fig. 3.**
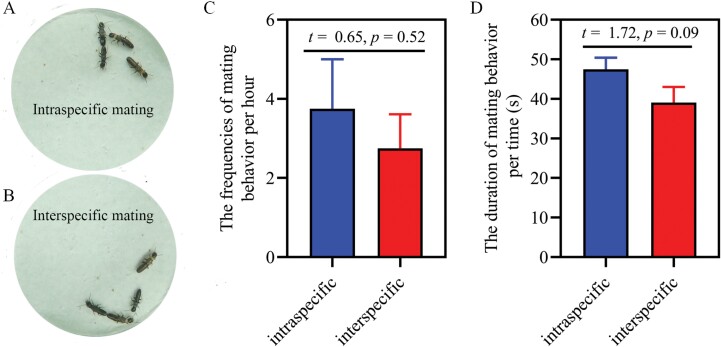
The frequencies and duration of mating in intraspecific and interspecific partners. A) Intraspecific mating behavior. B) Interspecific mating behavior. C) The frequencies of mating behavior. D) The duration of mating behavior. Data are shown as means ± SE.

We found that termites copulate in the opposite position by joining their abdominal ends (Fig. 3A and B) and multiple times copulate in post-pair formation (see Fig. 3A and Supplementary Video S1). When conspecific and interspecific female partners were present in the same arena, male of both *R. chinensis* and *R. flaviceps* were observed mating with conspecific female and interspecific female in a short interval time. The numbers of mating occurred in conspecific were equal to the mating occurred in heterospecific partners per unit of time (Fig. 3A; *t* = 0.65, *P* = 0.52). There were no significant differences in the duration of each mating between intraspecies and interspecies (Fig. 3B; *t* = 1.72, *P* = 0.09). These results indicated that the preferences for conspecifics were absent in mating behavior between *R. chinensis* and *R. flaviceps*.

Genotyping analyses of progeny showed that both heterospecific and conspecific mating in colony established by a *R. chinensis* couple and a *R. flaviceps* couple were able to produce living offspring (Table 1; for details on genotype, see Supplementary Table S2). Although the accurate proportion of offspring produced by hybridization and conspecific mating in colonies was unknown because of limited diagnostic alleles, we still propose that in the case of intraspecific mating being present in a colony, the interspecies mating can also produce living offspring in termites. Thus, the purpose of interspecific reproductive behaviors between *R. chinensis* and *R. flaviceps* may not be reproductive interference, but interspecies mating to acquire offspring.

## Discussion

Considering the life history of the entire termite colony, the reproductive pattern and relatedness of colony founders are closely linked to colony breeding structure, and both of them are considered to be major factors affecting the genetic structure of the population (Korb and Hartfelder 2008, Vargo and Husseneder 2009, Hartke and Baer 2011). A single colony is usually established by one female dealate (primary queen) and one male dealate (primary king) during swarm season, and then produces other colony members (Thorne et al. 1999, Korb and Hartfelder 2008). The relatedness of founders decides the genetic diversity of the colony because colony is a closed system throughout its life span with no additional genetic contributions (Hartke and Baer 2011). Deceased founders are replaced by their own descendant within the colony, and thus inbreeding certainly occurs between parents and their offspring (Kobayashi et al. 2013, Yashiro and Matsuura 2014). Although the special breeding model of AQS is present in some species (Matsuura 2017), the inbreeding between mother (primary queen) and son (secondary king) still could exist and reduce the genetic diversity of the colony. Moreover, only a few species have asexual reproduction. Thus, high genetic diversity in termites still mainly depends on the selection of partner and the establishment of colony.

The behavioral interaction among individuals plays a role in gene exchange among populations and may impede or facilitate gene exchange between species (Liu et al. 2007, Ritchie 2007). Our results showed that closely related species *R. chinensis* and *R. flaviceps* have similar reproductive behaviors and lack preferences for conspecifics when individuals from interspecies encounter each other during swarming. These results suggested that the hybridization between *R. flaviceps* and *R. chinensis* may be formed in nature. More importantly, hybridization can also produce living offspring in termites. The abundant genotypes produced by recombination in hybrids may facilitate higher genetic diversity than those of the parents.

Preference for conspecifics is an assortative mating behavior at the species level that individuals select in the light of corresponding genotypic or phenotypic traits (Harari et al. 1999, Shine et al. 2001, Slade et al. 2014, Li et al. 2015). It is considered one of the mechanisms to prevent gene interchange between species or dissimilative populations (Seehausen et al. 1997, Jiang et al. 2013). Previous studies suggested that preference for conspecifics is malleable, with the reproductive individuals accepting or rejecting an interspecific partner on the basis of environmental context (Pfennig 2007). For example, *Coptotermes gestroi* and *C. formosanus* in Florida field, the individual preference to tandem with conspecifics or heterospecifics can change depending on the arena (Chouvenc et al. 2015). However, in termites, the reproductive behaviors have extremely high risks, with plenty of alates randomly flying off their natal colony, but less than 1% individuals could successfully be paired and established a new colony due to sex ratio, predator, and other factors (Matsuura et al. 2002). Thus, rejecting an interspecific partner and seeking overzealously for an intraspecific partner may result in the loss of pairing opportunities. Failure to pair means loss of all fitness because the colony established by alone individual is mortal (Mizumoto et al. 2016). On the contrary, accepting a heterospecific partner permissively may or may not acquire offspring, but reproductive individuals will gain grooming opportunities and a longer lifespan. For termites, if partner resources in intraspecies are rare or predators exist, accepting a heterospecific partner may be a better choice. In our results, no preference for conspecifics suggested that heterospecific mating happens between 2 sympatric *Reticulitermes* species.

Interspecific mating will increase genotypes produced by recombination in hybrids and decrease inbreeding retrogression (Arnqvist 2011). It can also result in gene exchange between species and changes in genetic construction (Mallet 2005). However, independence was still maintained in some species when they have occurred interspecific mating and gene exchange. Similarly, there should be mechanisms to maintain the independence of species in termites due to the presence of hybridization, as a result of new speciation through hybridization is unusual. In termites, a colony was established by a couple, and gene exchanges with other colonies were limited by a closed nest (Thorne et al. 2002, Kobayashi et al. 2013). If hybrid offspring are fertile, then the replacement and backcross can be continued in hybrid colonies. The genetic dilution effect resulting from continuous backcross may eliminate genes of an original species and make the genetic construction of a hybrid colony come back to other original species’ gene pool. In other words, hybridization between species makes the genetic exchange; meanwhile, replacement and backcross resulting in a genetic dilution effect can prevent gene differentiation of hybrid colonies and then eliminate the possibility of hybrid speciation.

By mating with other species, the termites can acquire higher genetic diversity, increase the success rate of dealate reproduction, gain greater fitness, and retain the transmission rate of their genes to relative species. The inefficient preference for conspecific partners in congeneric species may also benefit from the genetic diversity of offspring because hybridization has higher genetic diversity compared to those produced by conspecific breeding.

## Supplementary Material

iead115_suppl_Supplementary_Tables_S1-S2

iead115_suppl_Supplementary_Videos_S1
