# Supplementary material for: Effect on genetic diversity of the absence of intraspecies preference in 2 sympatric Reticulitermes termite species (Isoptera: Rhinotermitidae)
Source: J Insect Sci. 2023 Dec 7;23(6):24. doi: 10.1093/jisesa/iead115 (PMC10701473; doi:10.1093/jisesa/iead115)
Supplement: iead115_suppl_Supplementary_Tables_S1-S2 [file iead115_suppl_supplementary_tables_s1-s2.docx]

**Supplementary Materials**

**Effect on genetic diversity of the absence of intraspecies preference in two sympatric *Reticulitermes* termite species (Isoptera: Rhinotermitidae)**

**Table S1** Primer sequences of the five microsatellite loci used in this study^*^.

| **Locus** | **Primer sequence 5′(r) 3′** | **Size (bp)** | **Ta (°C)** | **Repeat unit** |
| --- | --- | --- | --- | --- |
| Ra141 | F: CACATTTGAGGTTCGCAAGA  R: GCCAGAAGGCCAATTACAGA | 168 | 56 | (TTA)_8_ |
| Ra 144 | F: CAAATAGAGCTCCGTGTTTCG  R: CCATAGAAACCTCCGAAAGG | 158 | 55 | (TTAG)_7_ |
| Rs03 | F: TCCTGACTGTACAAAGAAAAGTGG  R: TGGCATCAAGCTACGTATTCA | 230 | 56 | (CT)_9_ |
| Rs76 | F: AATCCGGGGAATTTCTTGAC  R: CTGCATAACGATGTCTGCGT | 187 | 58 | (AGTT)_8_ |
| Rs78 | F: GCTTCTCAAGAAGGACTGTGC  R: GCCCCAGTTGAGATATGGAA | 178 | 58 | (AGTT)_7_ |

^*^Repeat units and sizes refer to the sequenced alleles. F, forward; R, reverse; Ta, annealing temperature (°C).

**Table S2** Genotypes of parents and larvae from the five colonies at the five microsatellite loci^*^.

| Colony ID | individual |  | SSR Loci^*^ | | | | |
| --- | --- | --- | --- | --- | --- | --- | --- |
|  |  |  | *Rs*78 | *Rs*76 | *Rs*03 | *Ra*141 | *Ra*144 |
| H*_cf_*_-032806_ | RC♀ |  | 177/188 | 169/174 | 238/238 | 188/203 | 155/159 |
|  | RC♂ |  | 172/188 | 174/187 | 232/238 | 201/103 | 155/159 |
|  | RF♀ |  | 164/177 | 162/182 | 232/238 | 188/201 | 155/155 |
|  | RC♂ |  | 188/188 | 169/174 | 238/238 | 195/195 | 155/159 |
|  | L1 |  | 164/188 | 162/174 | 238/238 | 188/195 | 155/155 |
|  | L2 |  | 172/177 | 162/174 | 238/238 | 201/103 | 155/159 |
|  | L3 |  | **172/188** | 162/174 | 232/238 | **203/203** | 155/159 |
|  | L4 |  | 164/188 | 162/174 | 238/238 | 201/203 | 155/155 |
|  | L5 |  | **164/172** | **162/187** | 232/238 | **195/201** | 159/159 |
|  | L6 |  | 188/188 | **162/187** | --/-- | 201/103 | 155/155 |
|  | L7 |  | 164/188 | 162/174 | 232/238 | 188/195 | 155/159 |
|  | L8 |  | 164/188 | 174/174 | 232/238 | 188/195 | 155/155 |
|  | L9 |  | 164/188 | 174/174 | 238/238 | **188/201** | 155/155 |
|  | L10 |  | 164/188 | **174/187** | 238/238 | 188/195 | 159/159 |
|  | L11 |  | 172/188 | **162/187** | --/-- | 188/195 | 155/155 |
|  | L12 |  | 172/188 | 162/174 | 238/238 | 188/195 | 155/159 |
|  | L13 |  | 172/177 | **162/187** | 232/238 | 195/195 | 155/155 |
|  | L14 |  | **172/188** | **174/187** | 238/238 | 188/195 | 159/159 |
|  | L15 |  | 172/177 | **174/187** | 232/238 | 188/195 | 155/155 |
|  | L16 |  | **172/188** | **174/187** | 238/238 | 188/195 | 155/159 |
| H*_cf_*_-032810_ | RC♀ |  | 164/172 | 162/174 | 232/238 | 188/195 | 155/155 |
|  | RC♂ |  | 177/181 | 169/174 | 232/232 | 195/197 | 155/155 |
|  | RF♀ |  | 164/172 | 178/182 | 232/238 | 195/195 | 155/159 |
|  | RC♂ |  | 172/181 | 169/174 | 238/238 | 188/201 | 155/159 |
|  | L1 |  | 164/172 | 162/174 | 232/232 | 188/195 | 155/155 |
|  | L2 |  | 172/177 | 174/174 | 238/238 | 195/201 | 155/159 |
|  | L3 |  | 164/172 | 174/174 | 238/238 | 195/201 | 155/155 |
|  | L4 |  | 172/177 | 174/182 | 232/232 | **188/201** | 155/159 |
|  | L5 |  | 172/177 | 174/174 | 238/238 | 188/195 | **159/159** |
|  | L6 |  | 172/177 | 162/174 | 238/238 | 188/195 | **159/159** |
|  | L7 |  | 172/181 | 174/178 | 232/232 | 195/201 | 155/155 |
|  | L8 |  | 164/172 | 162/174 | 232/232 | 188/195 | 155/159 |
|  | L9 |  | 172/177 | --/-- | 232/232 | 195/201 | **159/159** |
|  | L10 |  | 172/177 | 162/174 | 238/238 | 195/201 | 155/159 |
|  | L11 |  | 172/181 | --/-- | 232/232 | 188/195 | **159/159** |
|  | L12 |  | 172/181 | 169/174 | 238/238 | 188/195 | **159/159** |
|  | L13 |  | 172/181 | 169/174 | 232/232 | 188/195 | 155/159 |
|  | L14 |  | 172/177 | 162/174 | 238/238 | --/-- | 155/155 |
|  | L15 |  | 164/172 | 162/174 | 238/238 | **188/201** | 155/159 |
|  | L16 |  | 172/181 | 162/174 | 238/238 | **188/201** | 155/155 |
|  | L17 |  | 172/177 | 162/174 | 238/238 | **188/201** | 155/159 |
|  | L18 |  | 172/177 | 174/178 | 232/232 | --/-- | **159/159** |
|  | L19 |  | 172/177 | 174/178 | 232/232 | 188/195 | **159/159** |
|  | L20 |  | 164/172 | 174/178 | 238/238 | 188/195 | 155/155 |
| H*_cf_*_-032604_ | RC♀ |  | 164/172 | 174/174 | 238/238 | 188/195 | 155/163 |
|  | RC♂ |  | 164/177 | 169/174 | 238/238 | 195/195 | 155/155 |
|  | RF♀ |  | 164/177 | 162/174 | 232/232 | 188/188 | 159/159 |
|  | RC♂ |  | 164/172 | 174/187 | 232/238 | 188/195 | --/-- |
|  | L1 |  | 164/177 | 162/174 | 238/238 | 188/195 | 155/163 |
|  | L2 |  | 164/164 | **162/169** | 232/238 | 188/195 | 155/155 |
|  | L3 |  | 164/177 | **162/278** | 238/238 | 188/195 | 155/163 |
|  | L4 |  | **177/177** | 162/174 | 238/238 | 195/195 | 159/159 |
|  | L5 |  | 164/164 | 169/174 | 232/238 | 188/188 | 155/155 |
|  | L6 |  | 172/177 | **162/187** | 232/238 | 188/195 | 159/159 |
|  | L7 |  | 164/164 | 174/174 | 238/238 | 188/195 | 155/155 |
|  | L8 |  | 164/177 | 174/174 | 238/238 | 188/195 | 159/159 |
|  | L9 |  | 164/177 | 174/174 | **232/232** | 188/195 | 155/155 |
|  | L10 |  | **177/177** | 174/174 | 238/238 | 195/195 | 159/159 |
|  | L11 |  | 164/172 | 174/174 | 232/238 | 188/188 | 159/159 |
|  | L12 |  | 172/177 | 174/174 | 238/238 | 188/195 | 155/163 |
|  | L13 |  | --/-- | 174/174 | **232/232** | 188/195 | 155/155 |
|  | L14 |  | 164/177 | 174/174 | 238/238 | 188/188 | 155/155 |
| H*_cf_*_-032601_ | RC♀ |  | 177/177 | 174/174 | 232/232 | 195/201 | 155/159 |
|  | RC♂ |  | 164/177 | 162/174 | 232/238 | 195/195 | 155/159 |
|  | RF♀ |  | 164/172 | 162/174 | 238/238 | 188/201 | 155/155 |
|  | RC♂ |  | 177/181 | 174/174 | 232/238 | 195/195 | 155/159 |
|  | L1 |  | 164/177 | **162/162** | 238/238 | 188/195 | 155/155 |
|  | L2 |  | **164/181** | 174/174 | 238/238 | 188/195 | 155/159 |
|  | L3 |  | 164/164 | 162/174 | 232/232 | 188/195 | 155/159 |
|  | L4 |  | **164/181** | 174/174 | 232/238 | 195/201 | 155/155 |
|  | L5 |  | 164/177 | 174/174 | 238/238 | 195/201 | 159/159 |
|  | L6 |  | 164/177 | **162/162** | 238/238 | 195/195 | 155/155 |
|  | L7 |  | **164/181** | 174/174 | 232/238 | 195/201 | 155/159 |
|  | L8 |  | **164/172** | 174/174 | 238/238 | 195/195 | 155/155 |
|  | L9 |  | **164/181** | 162/174 | 232/232 | 195/201 | 155/155 |
|  | L10 |  | **177/181** | 174/174 | 238/238 | 195/201 | 159/159 |
|  | L11 |  | **177/181** | 174/174 | 232/238 | 195/195 | 155/155 |
|  | L12 |  | 177/177 | 174/174 | 238/238 | 195/201 | 155/155 |
| H*_cf_*_-032705_ | RC♀ |  | 165/177 | 171/183 | 232/242 | 193/202 | --/-- |
|  | RC♂ |  | 165/177 | 171/183 | 232/238 | 190/205 | 155/155 |
|  | RF♀ |  | 177/177 | 167/175 | 232/232 | 190/190 | 159/172 |
|  | RC♂ |  | 177/177 | 175/175 | 232/232 | 190/193 | 159/172 |
|  | L1 |  | 177/177 | **175/183** | 232/232 | **193/202** | 155/159 |
|  | L2 |  | 177/177 | **175/175** | 232/232 | 190/193 | 159/159 |
|  | L3 |  | 177/177 | **167/175** | 232/232 | 190/190 | 159/159 |
|  | L4 |  | 177/177 | **175/175** | 232/232 | 190/193 | 159/159 |
|  | L5 |  | 165/177 | **167/175** | 232/238 | 190/193 | --/-- |
|  | L6 |  | 177/177 | **167/175** | 232/232 | 190/193 | 159/159 |
|  | L7 |  | 177/177 | **167/175** | 232/232 | 190/193 | 159/159 |
|  | L8 |  | 177/177 | **167/175** | 232/232 | 190/190 | 159/159 |
|  | L9 |  | 177/177 | **171/175** | 232/232 | 190/190 | 155/159 |
|  | L10 |  | 177/177 | **175/175** | 232/232 | 190/190 | 159/159 |
|  | L11 |  | 177/177 | **175/175** | 232/232 | 190/190 | 159/159 |
|  | L12 |  | 177/177 | **171/175** | 232/232 | **193/193** | 155/159 |
|  | L13 |  | 165/177 | **175/183** | 232/242 | **193/193** | --/-- |
|  | L14 |  | 165/177 | **171/175** | 232/242 | 190/193 | 155/159 |
|  | L15 |  | 177/177 | **167/175** | 232/232 | 190/190 | 159/159 |
|  | L16 |  | 177/177 | **175/175** | 232/232 | 190/193 | 159/159 |
|  | L17 |  | 165/177 | **171/175** | 232/232 | 190/193 | --/-- |
|  | L18 |  | 177/177 | **167/175** | 232/232 | 190/190 | 159/159 |

^*^ Hybrid diagnosed loci were shown in red color. Pure-breed diagnosed loci were shown in blue color. Uncertain diagnosed loci were shown in black color. --/-- shows that the loci of this individual samples failed to PCR.
